# Focused Ultrasound Effects on Osteosarcoma Cell Lines

**DOI:** 10.1155/2019/6082304

**Published:** 2019-05-19

**Authors:** Valentina Agnese, Viviana Costa, Gian Luca Scoarughi, Cristiano Corso, Valeria Carina, Angela De Luca, Daniele Bellavia, Lavinia Raimondi, Stefania Pagani, Massimo Midiri, Giorgio Stassi, Riccardo Alessandro, Milena Fini, Gaetano Barbato, Gianluca Giavaresi

**Affiliations:** ^1^IRCCS Istituto Ortopedico Rizzoli, Via di Barbiano, 1/10 - 40136 Bologna, Italy; ^2^Promedica Bioelectronics srl, Dep. Research & Development, Via del Vespro, 129 - 90127 Palermo, Italy; ^3^Section of Radiological Sciences, University of Palermo, Via del Vespro 127 - 90127 Palermo, Italy; ^4^Section of Cellular and Molecular Oncology Section, Department of Surgical, Oncological and Stomatological Sciences, University of Palermo, Via del Vespro 131 – 90134 Palermo, Italy; ^5^Department of Biomedicine, Neuroscience and Advanced Diagnostics, Section of Biology and Genetics, University of Palermo, Via Divisi 83 – 90133 Palermo, Italy; ^6^Institute of Biomedicine and Molecular Immunology (IBIM), National Research Council, Via Ugo La Malfa 153-90146 Palermo, Italy

## Abstract

MRI guided Focused Ultrasound (MRgFUS) has shown to be effective therapeutic modality for non-invasive clinical interventions in ablating of uterine fibroids, in bone metastasis palliative treatments, and in breast, liver, and prostate cancer ablation. MRgFUS combines high intensity focused ultrasound (HIFU) with MRI images for treatment planning and real time thermometry monitoring, thus enabling non-invasive ablation of tumor tissue. Although in the literature there are several studies on the Ultrasound (US) effects on cell in culture, there is no systematic evidence of the biological effect of Magnetic Resonance guided Focused Ultrasound Surgery (MRgFUS) treatment on osteosarcoma cells, especially in lower dose regions, where tissues receive sub-lethal acoustic power. The effect of MRgFUS treatment at different levels of acoustic intensity (15.5-49 W/cm^2^) was investigated on Mg-63 and Saos-2 cell lines to evaluate the impact of the dissipation of acoustic energy delivered outside the focal area, in terms of cell viability and osteogenic differentiation at 24 h, 7 days, and 14 days after treatment. Results suggested that the attenuation of FUS acoustic intensities from the focal area (higher intensities) to the “far field” (lower intensities) zones might determine different osteosarcoma cell responses, which range from decrease of cell proliferation rates (from 49 W/cm^2^ to 38.9 W/cm^2^) to the selection of a subpopulation of heterogeneous and immature living cells (from 31.1 W/cm^2^ to 15.5 W/cm^2^), which can clearly preserve bone tumor cells.

## 1. Introduction

Magnetic Resonance guided Focused Ultrasound Surgery (MRgFUS), an image-guided non-invasive therapeutic treatment, is increasingly becoming popular for cancer ablation [1]. MRgFUS has also been recently adopted for the treatment of bone tumors, including benign tumors, primary malignancies, and metastatic bone tumors [2, 3]. In addition to the direct effect on bone cells, MRgFUS is widely used for palliative pain relief, thanks to its periosteal denervation action [4, 5]. Recently, Rodrigues et al. have analyzed results of fifteen clinical studies evaluating the effect of MRgFUS for non-invasive treatment of bone tumors at different levels of severity, showing a FUS efficacy of 92-100 %, 85-87 %, and 64-87 % for primary benign, primary malignant, and metastatic tumors, respectively [6]. However, other studies revealed that patients treated for primary malignant tumors have a higher risk of complications, highlighting that the question of FUS treatment safety is still under debate [1, 4, 7, 8]. In particular, the high acoustic impedance of cortical bone makes full ablation of bone lesions difficult and dangerous, suggesting the need of a better knowledge and control of ultrasonic interaction with cortical bone [9].

In MRgFUS the region of interest is targeted by high quality morphological MR images, which locally guides the FUS system application of High Intensity Focused Ultrasound (HIFU). MR images also ensure real time thermometry monitoring, thus enabling controlled and completely non-invasive ablation of tumor tissue [10]. The prime mechanism for HIFU cellular destruction due to local heating is largely understood; in fact locally delivered acoustic intensity generates a temperature rise above 55°C inducing proteins denaturation, cell death and resulting in local tissue coagulative necrosis [11].

Although the main lethal effect of HIFU is thermal, the deposition of energy on a target tissue may give rise to other relevant mechanical phenomena such as cavitation [13] and non-linear wave propagation [14–16]. The cavitation effect is a consequence of the interaction of acoustic waves with microscopic cavities containing vapour or gas disseminated into tissues or intracellular fluids, which can collapse and/or eventually result in bubbles of variable size under the action of the acoustic pressure force. The highly localized shear stress might cause cellular or even tissue damage [14]. The non-linear wave propagation effect, known as super harmonic leakage, originates when a large amplitude single frequency ultrasound wave travels through a non-linear medium. The waveform distorts and ultimately leaks energy from the fundamental frequency (transducer frequency) into higher harmonics; energy from these higher harmonics is absorbed by the tissue, in the near field and, at least partially, dissipated into heat. This non-linear effect becomes significant in case of treatment at increasing depth [11]. Hence, besides the thermal effect, all these effects may occur along the acoustic wave pathway, while crossing several layers of different tissues reaching the focal center. Conversely, once out of the focal area, the acoustic beam starts defocusing and diverging into the so-called “far field”. Attempts of understanding the effects of the near and far field on the tissue surrounding the focal volume have been phenomenological and aiming at improving the design of treatment [17]. Recently, attempts to correlate MRI findings to histological analysis following preclinical* in vivo* HIFU on animal models have been reported [18]. These were concentrating on comparing MR detection of precise contouring of the volume that has received a thermal dose sufficient to induce tissue death. However, attempts at understanding the effects of far or near-field interactions with tissue at the cellular levels are still lacking.

All the described modes of action may be active to some extent during FUS treatments on target tissue and have a variable effect both at the tissue and cellular level, such as extension of focal area [7], surrounding tissue damage [19], or metastatic spread [20, 21]. To the author's knowledge, very few studies have observed the biological and molecular effects of FUS treatment on osteosarcomas cells, especially in the lower dose region [19, 22]. Indeed, within a FUS ablative treatment, including volumetric type treatment, three different spatial zones can be identified: (i) the focal zone, where there is a sudden rise in temperature and thus an effective coagulative necrosis process; (ii) a second external zone, which surrounds the focal volume and whereby necrosis effects might still be induced by thermal drainage from the focal volume [23]; and (iii) a third more external zone, where the thermal drainage is not sufficient to induce necrosis. In these two more external transition zones, the tissues might be warmed to sub-lethal temperatures allowing a small percentage of cells, which might be cancer cells, to survive the thermal insult. If considering mechanical effects instead, these zones also roughly coincide with areas having received a lower, but still biologically relevant, dose of acoustic energy (near or far field).

Current authors recently investigated the mechanical transduction role of low intensity pulsed ultrasounds (LIPUS) on different* in vitro* cell models (tumor or normal cells), finding that they are able to reduce osteolytic ability of breast cancer cell (*under review*) and to induce pre-osteoblast commitment and differentiation [24].

The aim of the present study was to mimic* in vitro* the response of cells receiving an ultrasound energy dose at the “far field”, comparable to that which would be received by cells within tissues located in external zones in the range of 5-30 mm from the focal volume. A specific experimental set-up was realized to verify the mechanical (cavitation and non-linear wave propagation) effect of the FUS treatment on osteosarcoma cell lines. To this purpose, FUS treatments at different dose levels of acoustic intensity, inversely proportional to the distance from the focus, were applied on Mg-63 and Saos-2 cell lines. Cell viability and expression levels of osteoblastic markers were analysed at different time points after the stimulation.

## 2. Materials and Methods

### 2.1. Acoustic Pathway Setup for MRgFUS on Multi-Well

**Figure 1:**
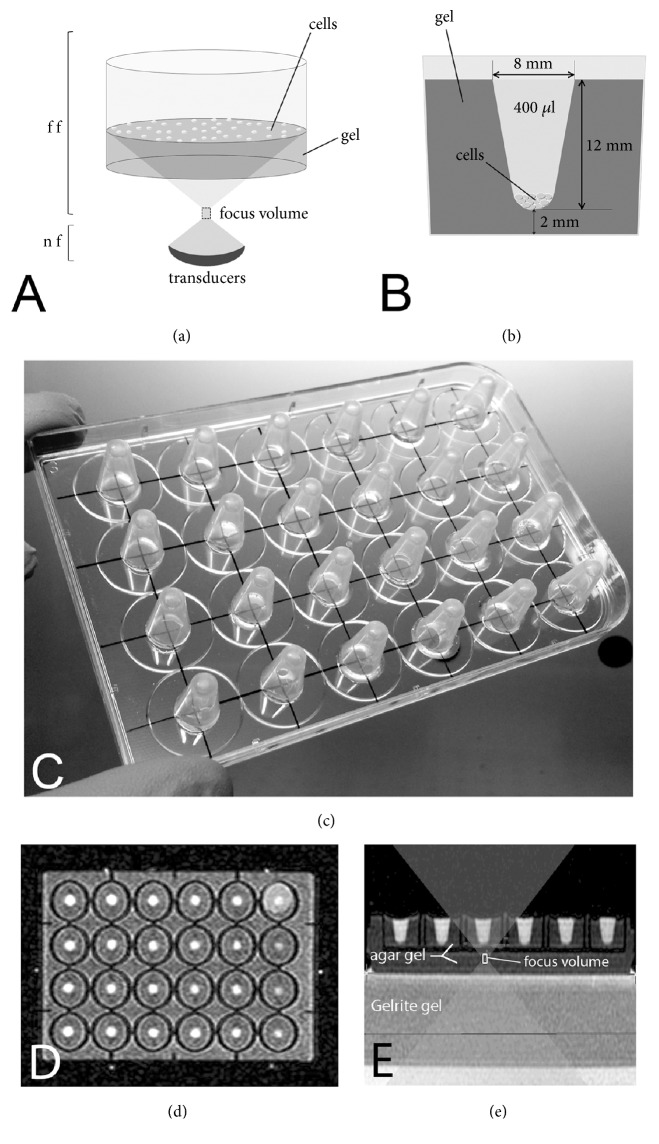
Multiwell plate setups analysed in MRgFUS experiments: (a) Flat-setup where 24 wells were previously filled with 0.5 ml of agar gel (the figure is not in scale: the transducer array is depicted nearer to the well and smaller than in the reality); (b) V-setup, where the wells were previously filled with 2.5 ml of agar gel and a V-bottom shape was realized applying a modified 24 polystyrene lid (c) on the plate until the agar had solidified. In the V-setup, cells were concentrated in a reduced volume for better uniformity of exposure to the acoustic field. (d) and (e) images represent the MR axial and coronal sections, respectively, of the multiwell plate prepared with the V-setup. Note that panel schemes are not in scale.

The ExAblate 2100 system (lnSightec Ltd, Israel) was used in combination with a MRI System (1.5 Tesla GE, USA), for sonications of 24 multi-well polystyrene plates (24-well plates, Corning, NY, USA). A 208-element phased array transducer, with a 160 mm radius of curvature and a 120 mm diameter and used at the operating ultrasound frequency of 1.05 MHz, was immersed into a mylar sealed circular degassed and cooled bath of water inside the MRgFUS patient table. The overall range of acoustic delivered to the target explored was 15.5-49 W/cm^2^. In order to verify that the data reported from the ExAblate software about the acoustic energy delivered to the target were correct, the system was calibrated using the radiation force balance method. Several sonications were performed using an absorber target; the acoustic pressures were reported by a precision weight scale (CM150-1N, KERN & Sohn GmbH, Germany). The shifts between the data collected and the ones reported from the ExAblate software were always under the 2%. The focus of the generated field is generally described in terms of a cylindroid shape whose boundaries describe an iso energetic surface obtained at -6 dB. In our case the cylindroid has an approximate size of 9 × 3 mm, with the longer vertical axis oriented parallel to the axis of the plate-well (in Figure 1(e) symbolized by a small rectangle). In the following, all references to the focal point are meant as distance from the top of the cylindroid surface.

To acoustically couple the transducer source and the modified multi-well, we used intermediate gel layers positioned between the mylar seal and the multi-well. To this purpose high water content gels were prepared: the gel layer was cylindrical in shape and contained within a homemade larger plexiglass cylinder with a thin bottom and an inlet and outlet valve for continuously circulating and degassing water from a 38°C thermostated bath. Several gelling polymers were considered (agar, agarose, Gelrite™) and different concentrations from 1.25 % to 4 % were explored. Final gel parameters to record data were 1.4 % Gelzan™, 210 mm diameter, 43 mm height. The gel layer proved effective also in thermally decoupling the multi-well from the refrigerated bath containing the transducer.

The advanced mode of InSightec clinical software was used to interface the MR and FUS system and to plan the dimension and the position of the focal area and the acoustic energy of the ultrasound wave. The focus location accuracy was verified before the experiment using tissue mimicking phantom and MRI thermometry. The top of the focal volume longitudinal axis was parallel to the central z-axis of each well, and its top was 5 mm below the cell layers (measured on the MR image with a scale built in the InSightec's software) (Figure 1(e)). This set-up ensures that the front of ultrasonic waves delivering acoustic energy belongs entirely to the Far Field, allowing, according to the acoustic wave propagation theory [25], a homogeneous delivery of ultrasound to cells into each well. Cavitation occurrences were kept under control using the ExAblate 2100 built-in cavitation detector, based on spectrum measurement [26]. Lab-grade degassed water was always used.

Modified multiwell plates were prepared under sterile condition as follows: the inner and outer cavities, except wells, were filled by agar gel (agar 2.25%, NaCl 0.9%); this procedure eliminates air/liquid interfaces as potential sources of refraction and reflection for the acoustic waves. The same gel was also employed to make an extra bottom layer, 10 mm thick, which prevented air-bubbles from trapping between the phantom and the multiwell. Gel casting procedures were made on planar surfaces. Two multiwell setups were initially explored: in the flat-setup (Figure 1(a)) the wells were previously filled with 0.5 ml of agar gel (agar 2.25%, NaCl 0.9%), while in the V-setup (Figure 1(b)) the wells were previously filled with 2.5 ml of agar gel (agar 2.25%, NaCl 0.9%) and a modified 24 well polystyrene lid was placed on the 24 multiwell plate until that the agar had solidified. The modified lid had 24 polypropylene cones glued in the centre of each well axis (Figure 1(c)). After the gel had completely solidified and the lid had been removed, the centre of each well had a 400 *μ*l conical shaped cavity. The modified lid was routinely sterilized by a step of immersion for 5 min in a 24 multiwell filled with ethanol 70% followed by 15 min of UV exposure.

The V-setup was preferred to the flat-setup because it concentrates the cells in a reduced volume with respect to the flat setup (when using single element flat transducers, for a more uniform US delivery to cells, the flat set-up should be used instead). The distribution of cells into the wells is depicted in Figures 1(a) and 1(b). The MR axial (Figure 1(d)) and coronal (Figure 1(e)) views of the plate-well are reported, evidencing also the acoustic cone and the focus position, with respect to the Z coordinate of the plate.

### 2.2. Thermal and Acoustic Calibrations inside the Well

To optimize the focal volume positioning with respect to the well bottom, and in order to avoid undesired thermal effect propagation we performed simulations to establish where and to which extent the acoustic energy would be dissipated thermally within the gel loaded wells, so that we could concentrate our thermal energy decoupling efforts to those regions.

**Figure 2:**
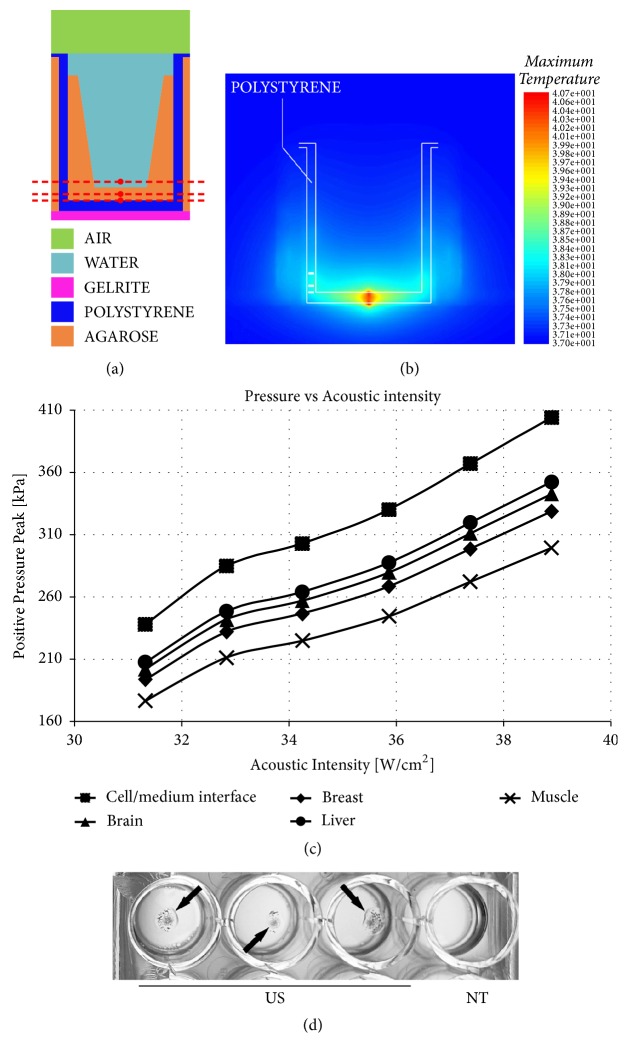
Thermal and Acoustic calibrations: (a) Target V-setup model used in simulations with PZFlex® software. The spot in each dotted red line shows where the thermocouple was placed during the thermal calibration; (b) Thermal map obtained from simulation:49 W/cm^2^ irradiated for 10 s produce a 40.7°C hot spot, localized inside the polystyrene 24-well bottom. (c) Acoustic graphs. Following the theory described by Laugier [12], along the acoustic axis the pressure decays with an exponential trend is given by: *p* = *p*_0_*e*^−*αz*^ where *α* is the attenuation coefficient, *p*_0_ the pressure at the reference distance,* p* the pressure at the desired distance and z the difference between reference and desired distance. “Cell/medium interface” is the positive pressure peak measured by hydrophone at the interface agar-water inside the well during a 10 s sonication with an acoustics intensity range 31.3-38.9 W/cm^2^. “Brain”, “Breast”, “Muscle” and “Liver” are the estimated value at the top of the well for an equivalent relative volume of homogeneous tissue crossed. The estimated curve for water has not been shown since it was essentially overlapping with the experimental curve. The *α* values (dB/Mhz·cm) used were: water = 0.0022; brain = 0.6; breast = 0.75; liver = 0.5; muscle = 1.09 (Culjat, et al. 2010); (d) thermal damage of the polystyrene layer at the bottom of a 24-well multiwell plate (arrows). The US focus was placed inside the well, 1 mm from the bottom. Starting from left, the first three wells were treated with the same acoustic energy, while the last remained untreated. Even the lowest acoustic energy adopted in this study resulted in a visible damage of the polystyrene.

The MediFlex toolkit of PZFlex® software (Weidlinger Associates Inc, CA, USA) was used to simulate the physical effects produced by ultrasound (Figures 2(a) and 2(b)). The simulations were conducted assuming a single element focused transducer with the same aperture and radius of curvature of the array used in the ExAblate 2100 system (Figures 2(a) and 2(b)).

Calibration experiments on gel loaded wells were performed accordingly where the readouts were the temperature and the acoustic pressure, so as to verify that effective uncoupling from thermal effects was indeed obtained. Furthermore, we measured the acoustic field inside the well to have a measure of how homogeneous was its propagation within the well. To obtain the setup shown in Figure 1(a), the focal region was placed inside the Gelrite™ and the distance from the top of the focus to the cells was 5 mm. Experimental readings were performed with a set of 3 readouts of temperature using an 8-channel Data Logger OM-CP-OCTTEMP-A (Omega, Manchester, UK) equipped with 3 Type-T 0.5 mm thermos couples in copper-constantan. Measurements were taken outside the MRI, placing the thermocouples tip along the acoustic axis at 3 distances referred to the top surface of the gel where the cell layer is positioned (0 mm): +1, -1 and -2 mm, red dashed lines in Figure 2(a).

Acoustic pressure measurements on the multiwell solution have been carried out using 0.5 mm needle hydrophone (Precision Acoustic, UK). The tip of the hydrophone was positioned at the cell layer immediately above the culture medium/gel interface (Figure 2(a)). The hydrophone was moved using a handmade fixed position holder. The target was exposed to US for 10 s each time using an acoustic intensity in the range 15.5-49 W/cm^2^. The hydrophone was provided with a submersible preamplifier, 8 dB of nominal gain, and coupled to an oscilloscope DSOX3104A (Agilent Technologies Co Ltd, CA, USA). Readouts were in Volts and converted in pressure units considering the hydrophone sensitivity, 356 mV/MPa at 1.05 MHz. To asses dampening to the acoustic propagation for each element within the pathway, measures were performed in the presence and in the absence of one of the pathway elements. For example, a comparison was made between the pressures detected by the hydrophone in the target when acoustic waves pass through the experimental pathway (transducer-water-mylar-gelrite-polystyrene-agar-target), and at the same distance from the transducer without the experimental pathway (transducer-water-mylar-water-target). For each measurement the data collected at the same acoustic intensity were overlapped, also considering the hydrophone precision.

One of the most important differences between* in vitro* and* in vivo* setups is the medium-caused ultrasound field intensity attenuation.* In vitro* medium is either water or very hydrated gel, while* in vivo* medium is represented by different tissues. In Figure 2(c) we compare the pressure measured within the well at the bottom (gel-culture media interface) and at +12 mm from this point with the expected decay estimated for tissues.

### 2.3. Human Osteosarcoma Cell Lines and Culture Medium Components

The human osteosarcoma cell lines, Mg-63 and Saos-2, were purchased from ATCC (USA) and cultured in Dulbecco's modified Eagle medium (DMEM, Gibco BRL, Gaithersburg, MD) supplemented with 10 % fetal bovine serum (Gibco BRL), penicillin and streptomycin (100 U/ml, Gibco BRL), and fungizone (0.25 *μ*g/ml, Gibco BRL) at 37°C in 95 % air / 5 % CO_2_-humidified atmosphere. The culture medium was changed every 3 days and cells were split at 80–90 % of confluence using StemPro Accutase (GibcoBRL).

### 2.4. In Vitro FUS Treatment

Twenty-four hours before FUS treatment, Mg-63 and Saos-2 cells were seeded in V-setup plates (V-shaped inner volume 400 *μ*L, gel height from well bottom 2 mm, thickness of well bottom 1.2 mm) at a concentration of 70.000 cells/well. Shared settings were the patient's bed, containing the US transducer array immersed in coolant, and an upper mylar circular window transparent to ultrasound on which the phantom was placed.

Each well was filled with culture media and sealed by a gas-permeable adhesive film without air-bubble trapped inside. During sonications a sound-absorbing material, Aptflex F28, 10 mm thickness (Precision Acoustics, UK), was placed over the plate to minimize reflections.

Cell cultures were divided in 8 groups according to different operating acoustic energies: 0 (Control - cells that were handled in the same way as the treated ones except for the FUS treatment), 15.5, 23.3, 31.1, 38.9, 41.2, 46.6, and 49 W/cm^2^ (20, 30, 40, 50, 53, 60, and 63 W for 10 s sonication). Then, the V-set up plates were placed on the MRgFUS system and each well was exposed to the relative acoustic energy for 10 seconds, the cooling and repositioning times were 50 s/well, and the duration of the FUS treatment for an entire V-setup plate lasted 20 min. Each experimental condition was set up in triplicate. At the end of the single FUS stimulation, plates were cultured at 37°C in 95 % air / 5 % CO_2_-humidified atmosphere at three different experimental times: 24 h, 7, and 14 days. At the end of each experimental time point, cells were collected, centrifuged at 1200 rpm for 5 min, and split in two fractions: one half was used to evaluate cellular viability and the second fraction to evaluate osteogenic genes expression.

### 2.5. Cell Viability Assay

The proliferation of osteosarcoma cell lines was evaluated by a CellTiter-Glo Luminescent Cell Viability Assay (Promega Italia Srl, Milan, Italy). Briefly, 100 *μ*l of Mg-63 or Saos-2 cell suspensions in culture medium was transferred in a 96-well plate. The same amount of CellTiter-Glo reagent was added to each well. After 10 min of incubation at room temperature, ATP produced by metabolic active cells was quantified by luminescence emission detected by a Clariostar microplate reader (BMG LABTECH GmbH, Ortenberg, Germany) and expressed as Relative Luminescence Units (RLU) produced from viable cells. Cell viability results were reported as relative fold (RF) of FUS untreated culture (0 W/cm^2^).

### 2.6. RNA Extraction and Complementary DNA Synthesis

Total RNA was extracted with the use of the PureLink™ RNA Micro Kit (Invitrogen™, Life Technologies Italia–Monza, Italy) according to the manufacturer's instructions. After evaluation of amount and integrity by Nano Drop assay protocol (Thermo Fisher Scientific Inc., Fisher Scientific Italia, Rodano-Milan, Italy), total RNA was reverse transcribed with a High Capacity cDNA Archive kit (Applied Biosystems™, Life Technologies Italia – Monza, Italy) according to the manufacturer's instructions, to obtain complementary DNA (cDNA). Each cDNA sample was tested in duplicate.

### 2.7. Quantitative Polymerase Chain Reaction (RT-qPCR) Analysis

RT-qPCR was carried out with StepOne™ Real-Time PCR System (Applied Biosystems™) using SYBR® Green Real-Time PCR Master Mix (Applied Biosystems™). The following custom-made primers (Invitrogen™) were used for the detection of osteoblast differentiation: runt-related transcription factor-2 (*RUNX2*) Hs_RUNX_l_SG, alkaline phosphatase Hs_ALP_1_SG (*ALPL*) and osteocalcin (*BGLAP*) Hs_Osteocalcin_1_SG. The comparative Ct method was used to quantify relative gene expression with the formula 2^−∆∆Ct^, against* GAPDH* as reference gene and untreated Mg-63 and Saos-2 as calibrators at each experimental time point.

### 2.8. Statistical Analysis

Statistical analysis was performed using the IBM® SPSS® Statistics 23 software. Data are reported as median (Mdn) and median absolute deviation (MAD) [27]. After having verified that data were not normally distributed (Kolmogorov-Smirnov test), Kruskal–Wallis test by ranks, followed by Mann-Whitney pairwise comparisons using Bonferroni correction, was done to compare data between FUS acoustic intensities within each experimental time (for cell viability between FUS acoustic intensities and Control) and between experimental times within each FUS acoustic intensity.

## 3. Results

### 3.1. Temperature and Acoustic Measurements

Several simulations and measurements were carried out to ensure that the biological effects caused by ultrasonic wave were mainly due to mechanical, rather than thermal stress (Figure 2(b)). We were able to show a temperature increase, localized within the polystyrene layer at the bottom of the well, which would damage this layer when the focus was moved to the well medium, 1 mm from the bottom of the well (Figure 2(d)). A critical point is the thin polystyrene interface where temperature increases due to the different acoustic properties of polystyrene (would act as a sink of heat) locally enhancing the layer temperature and radiating a non-even gradient of temperature within the medium. Thus, we searched for optimal conditions where no thermal effects could be transmitted from the heating of the polystyrene bottom. The most favorable results were found when the top of the focal volume was placed about 2 mm externally, below the polystyrene bottom of the plate in the highly hydrated gel, at about 5 mm from the gel adhered cell layer inside the well. For example, when an acoustic intensity of 38.9 W/cm^2^ was delivered to the target (top of focal volume at -5 mm from cell layer), the simultaneous readouts were 40.2°C at -2 mm (tip in contact with the inner part of the bottom well), 38.7°C at -1mm (in the center of the gel) and 36.9°C at +1 mm (above gel-culture medium interface).

When an acoustic field generated by the MRgFUS crosses a tissue, the resulting intensity along the direction of propagation of the field will be a dampening of intensity caused by two factors: (i) the decay in intensity due to the defocusing of the field as it propagates away from the focus and (ii) the attenuation coefficient of each of the tissues crossed. The former factor is present in both the* in vivo* and in the* in vitro* applications. The latter, however, might be rather different for the two applications.* In in vivo*, considering some human tissues with their attenuation coefficients [28], the estimated acoustic pressure decayed over +12 mm space ranges from 12.3 % up to 25.8 % (Figure 2(c)). On the contrary,* in in vitro* by considering the volume of medium contained in a well, the acoustic pressure decayed less than 0.1 % in water along the same 12 mm pathway, going from the bottom (cell-medium interface) to the top of the medium within the well. Therefore, to simulate the* in vivo* type decay in intensity, each experiment was repeated with a different power setting, ranging from 15.5 to 49 W/cm^2^.

Dispersion and leakages were minimized with the final set-up described in Figure 1; an optimal acoustic pathway was established starting from transducer vibrating element crossed in the following order: water-mylar-highly hydrated gel-polystyrene bottom plate-highly hydrated gel-cell layer-medium-polyethylene adhesive film-acoustic gel-acoustic adsorber. We hypothesized that the gel present below and above the polystyrene layer should be less effective than the aqueous medium in diffusing thermal energy, and thus acting as thermal decoupler for the medium (Figures 1 and 2). To verify this hypothesis the effects of sonication on local temperature were monitored. MRgFUS* in vivo* has the advantage of measuring the temperature variation while sonicating [10]. Continuous monitoring allows the real time evaluation of the temperature in the focal region within the target tissue. However, this method relies on the slow diffusion rate of proton containing molecules (usually water) originating the MR signal monitored within the tissue being ablated. In our experiments the fast water diffusion kinetics within the well solution prevented us from obtaining a very precise temperature measurement (actual average STDEV range ±2-3°C,* data not shown*) and was similar to what has been previously reported in the literature [29]. Even at high acoustic intensity (i.e. 46.6 W/cm^2^) the thermal oscillations around the portion of gel position, where the cell layer was located, were found to be less than 2°C, going from 38.7°C at – 1 mm to 36.9°C at + 1 mm, where distances are referred to the adhesive cell layer/medium interface.

Our simulations and the measurements of acoustic pressure within the plate well using different energy levels exhibited an almost constant value of pressure along about 400 *μ*l of solution, evidencing a decay from the cell layer adhered on the gel to the top of the well (about 14 mm) of less than 0.1 %, although not representative of what would be found in human tissues. We thus mimicked the functional effect induced by decay exploring a range of acoustic intensities (15.5-49 W/cm^2^) that encompassed the levels that would be experienced by tissues located “for example” at 5-20 mm away from the focal point.

### 3.2. Cell Viability

**Figure 3:**
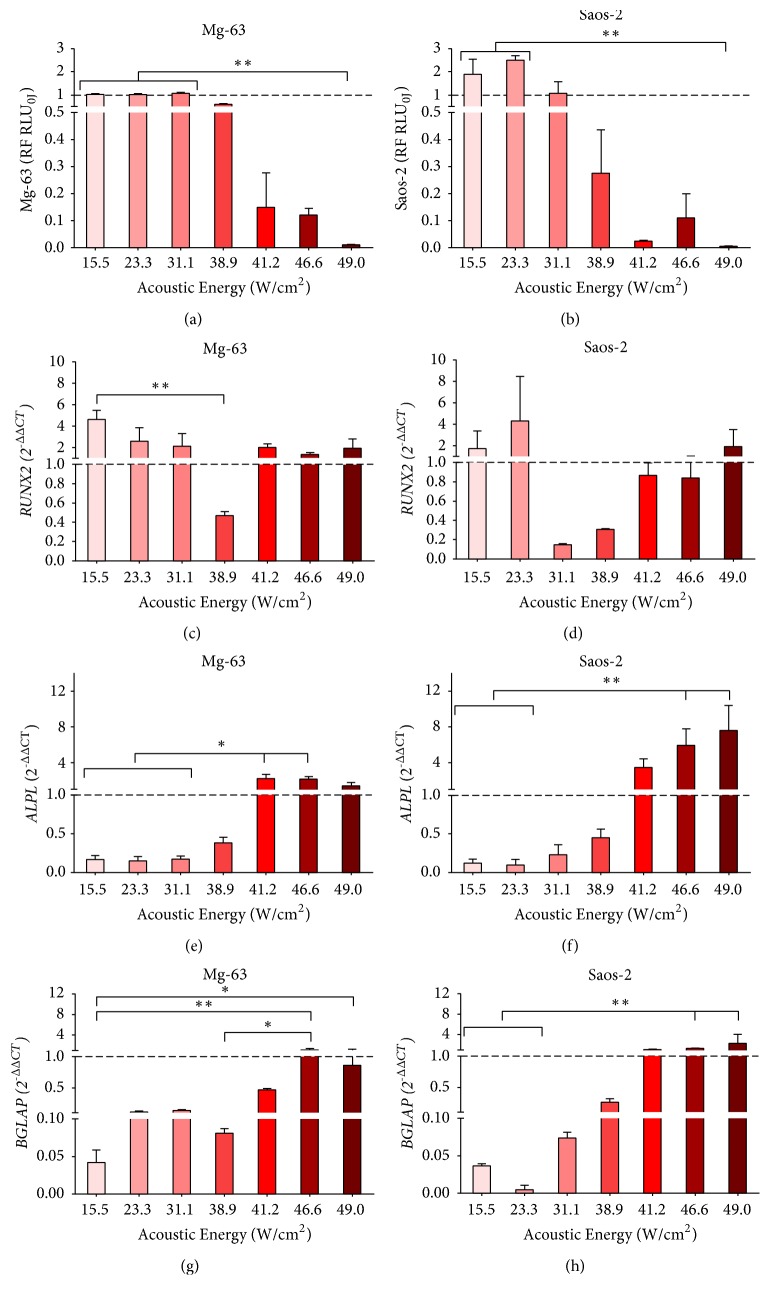
Cell viability ((a) and (b)) and gene expression of* RUNX2* ((c) and (d)),* ALPL* ((e) and (f)) and* BGLAP* ((g) and (h)) in Mg-63 (a, c, e, g) and Saos-2 (b, d, f, h) cultures at 24 h after FUS treatment with different acoustic intensities. Mdn ± MAD (n = 4 replicates). For each cell type, cell viability was expressed as RF of untreated culture (0 W/cm^2^) and gene expression as 2^−∆∆CT^, using gene expression of FUS untreated culture as calibrator. Mann Whitney U test between FUS intensities (*∗*,* p <* 0.05, *∗∗*,* p <* 0.005, *∗∗∗*,* p *< 0.0005).

**Figure 4:**
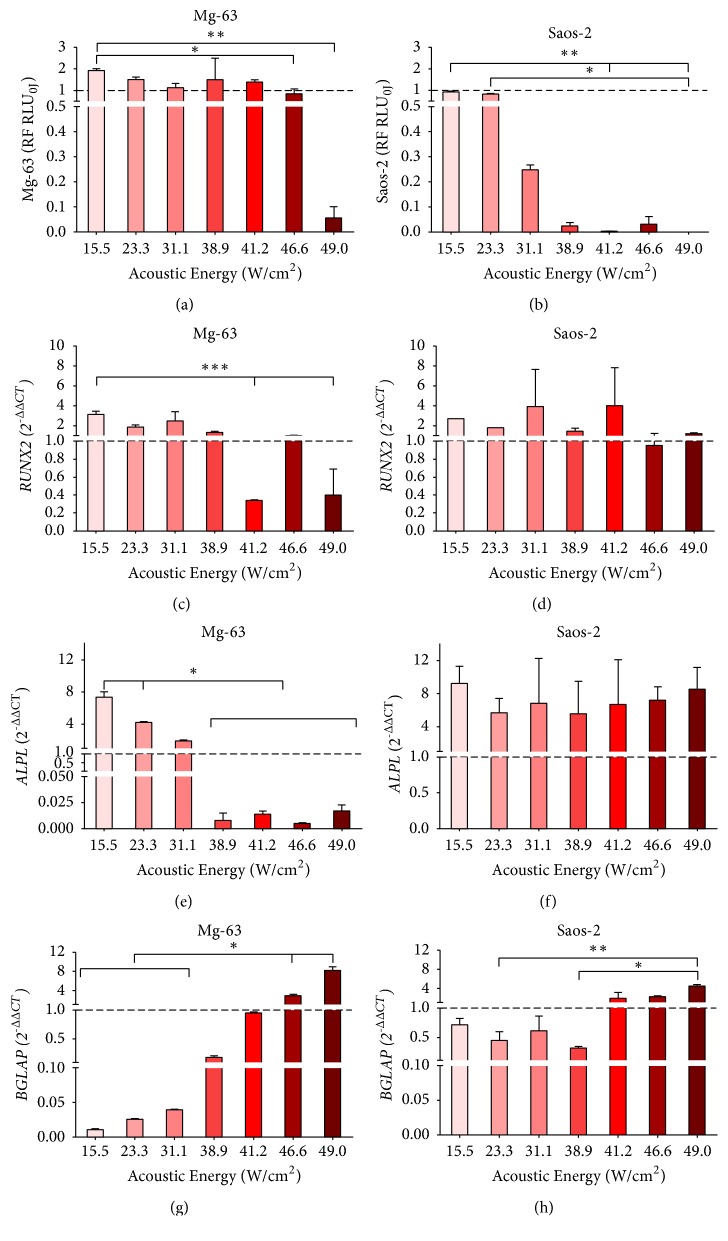
Cell viability ((a) and (b)) and gene expression of* RUNX2* ((c) and (d)),* ALPL* ((e) and (f)) and* BGLAP* ((g) and (h)) in Mg-63 (a, c, e, g) and Saos-2 (b, d, f, h) cultures 7 days after FUS treatment with different acoustic intensities. Mdn ± MAD (n = 4 replicates). For each cell type, cell viability was expressed as RF of untreated culture (0 W/cm^2^) and gene expression as 2^−∆∆CT^, using gene expression of FUS untreated culture as calibrator. Mann Whitney U test between FUS intensities (*∗*,* p <* 0.05, *∗∗*,* p* < 0.005, *∗∗∗*,* p *< 0.0005).

**Figure 5:**
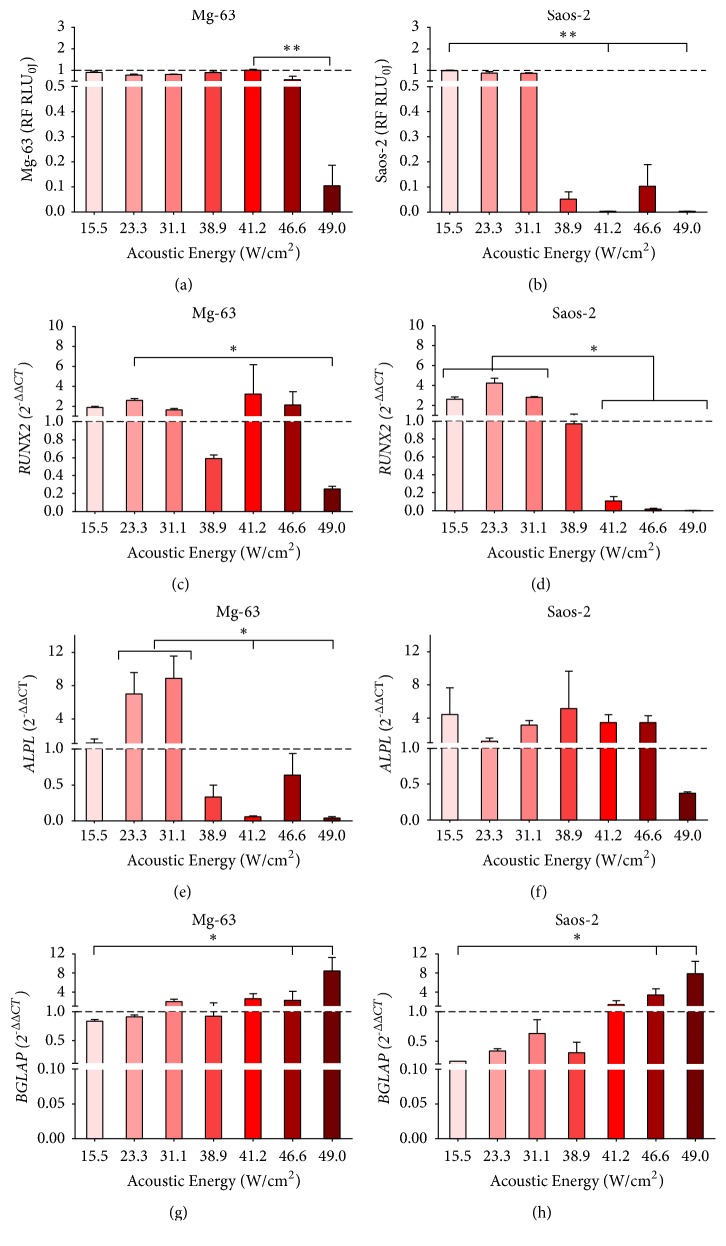
Cell viability ((a) and (b)) and gene expression of* RUNX2* ((c) and (d)),* ALPL* ((e) and (f)) and* BGLAP* ((g) and (h)) in Mg-63 (a, c, e, g) and Saos-2 (b, d, f, h) cultures 14 days after FUS treatment with different acoustic intensities. Mdn ± MAD (n = 4 replicates). For each cell type, cell viability was expressed as RF of untreated culture (0 W/cm^2^) and gene expression as 2^−∆∆CT^, using gene expression of FUS untreated culture as calibrator. Mann Whitney U test between FUS intensities (*∗*,* p <* 0.05, *∗∗*,* p *< 0.005, *∗∗∗*,* p* < 0.0005).

The results of cell viability and gene expression of Mg-63 and Saos-2 exposed to the different FUS acoustic intensities are reported in Figures 3, 4, and 5 for each experimental time.

When Mg-63 cells were exposed to FUS acoustic intensities ranging from 38.9 W/cm^2^ to 49 W/cm^2^, their viability decreased 24 h after treatment, down to be almost null with 49 W/cm^2^ (*p <* 0.005 in comparison to 15.5, 23.3, and 31.1 W/cm^2^) (Figure 3(a)). At 7 days, Mg-63 cells showed higher viability values with a significant decrease at 46.6 W/cm^2^ (*p <* 0.05) and 49 W/cm^2^ (*p <* 0.005) in comparison with 15.5 W/cm^2^ (Figure 4(a)). At 14 days, Mg-63 cell viability slightly decreased with each FUS acoustic energy compared to untreated culture, and the lowest cell viability result was achieved at 49 W/cm^2^ (*p <* 0.005) in comparison with 41.2 W/cm^2^ (Figure 5(a)).

Regarding Saos-2 viability at 24 h, this showed a trend similar to Mg-63 (Figure 3(b)); when the acoustic intensity was 38.9 W/cm^2^ or higher, Saos-2 cell viability decreased compared to the untreated group, with the lowest value at 49 W/cm^2^ (*p <* 0.0005) in comparison to 23.3 W/cm^2^. After 7 days from treatment, Saos-2 cell viability was lower than the control at all acoustic intensities, reaching very low levels from 38.9 W/cm^2^ (Figure 4(b)). Significant differences were found at 41.2 W/cm^2^ (*p <* 0.05) and 630J (*p <* 0.05) compared to 15.5 W/cm^2^ and at 49 W/cm^2^ compared to 23.3 W/cm^2^ (*p <* 0.005). Saos-2 cell viability of at 14 days was similar to that at 7 days, with lower values observed at 41.2 W/cm^2^ (*p < *0.005) and 49 W/cm^2^ (*p < *0.005) compared to 15.5 W/cm^2^ (Figure 5(b)).

Current data could suggest that the exposure of Mg-63 and Saos-2 cell lines to FUS low acoustic intensities (15.5-31.1 W/cm^2^) does not cause any significant variation in terms of cell proliferation.

### 3.3. Gene Expression

To investigate if the exposure to different FUS acoustic intensities could have an effect on the molecular pathways governing osteoblastic differentiation, we analyzed the variation of expression levels of* RUNX2, ALPL, *and* BGLAP* in Mg-63 and Saos-2 cell lines treated with FUS. Specifically,* RUNX2 *(a master osteoblast transcriptional factors) and* ALPL* play a critical role in the regulation of early phases of osteogenesis, whereas* BGLAP* is associated with a more mature osteogenic differentiation [30–32].

Twenty-four hours after FUS treatment, Mg-63 cells showed higher* RUNX2* expression levels compared to the untreated culture in all experimental condition except with 38.9 W/cm^2^, which showed a significant decrease of expression compared to 15.5 W/cm^2^, (*p <* 0.005) (Figure 3(c)). Saos-2 cultures showed high* RUNX2 *expression with 15.5, 23.3, and 49 W/cm^2^ without any significant difference compared to other FUS acoustic energies, which presented* RUNX2* values lower than untreated group (Figure 3(d)). At 7 days,* RUNX2 *was more expressed in Mg-63 cultures than in untreated culture, except for those treated with 41.2 and 49 W/cm^2^acoustic intensities, resulting in significant values lower than 15.5 W/cm^2^ (*p *< 0.05) (Figure 4(c)). Similarly,* RUNX2* expression was higher in Saos-2 treated than in untreated cultures, except for that at 46.6 W/cm^2^, without significant differences among FUS acoustic intensities (Figure 4(d)). Even at 14 days,* RUNX2* was more expressed in Mg-63 treated cultures, except for those exposed to 49 W/cm^2^ (*p <* 0.05) compared to 23.3 W/cm^2^ (Figure 5(c)). On the contrary, at 14 days* RUNX2* was less expressed in Saos-2 treated with FUS acoustic energies from 41.2 to 49 W/cm^2^ than those treated with 15.5, 23.3 and 31.1 W/cm^2^ (*p <*0.05) (Figure 5(d)).

*ALPL* expression levels increased proportionally to the increase of FUS acoustic intensity at 24 h in both cell types (Figures 3(e) and 3(f)): Mg-63 cultures showed a significant (*p <* 0.05) upregulation at 41.2 and 46.6 W/cm^2^ in comparison to 15.5, 23.3, and 31.1 W/cm^2^, while for Saos-2 this was evident (*p *< 0.005) at 46.6 and 49 W/cm^2^ in comparison to 15.5 and 23.3 W/cm^2^. At 7 days, the expression of* ALPL* in Mg-63 was upregulated compared to untreated culture up to 31.1 W/cm^2^ and strongly downregulated between 38.9 and 46.6 W/cm^2^ (*p <* 0.05), when compared to those observed at 15.5 and 23.3 W/cm^2^ (Figure 4(e)). On the contrary,* ALPL *expression results were always upregulated in Saos-2, without significant differences among FUS acoustic intensities (Figure 4(f)). At 14 days, Mg-63 cultures presented significant (*p <* 0.05) higher* ALPL *levels at 23.3 and 31.1 W/cm^2^ in comparison to lower levels observed at 41.2 and 49 W/cm^2^ (Figure 5(e)). No significant differences were observed for* ALPL *expression in Saos-2 at 14 days (Figure 5(f)).

In all experimental times and in both cell lines, the expression of* BGLAP* was downregulated up to an acoustic intensity of 41.2 W/cm^2^ and upregulated at higher intensities in comparison to the untreated cultures. At 24h,* BGLAP* was downregulated in Mg-63 cultures, except with 46.6 W/cm^2^, where a significantly higher value in comparison to 15.5 W/cm^2^ (*p* < 0.005) and 38.9 W/cm^2^ (*p *< 0.05), and 49 W/cm^2^ in comparison with 15.5 W/cm^2^ (*p <* 0.05) was observed, without being higher than untreated culture (Figure 3(g)). In Saos-2 cultures,* BGLAP* slightly increased with 46.6 W/cm^2^ and 49 W/cm^2^, showing a significantly higher value compared to 15.5 W/cm^2^ and 23.3 W/cm^2^ (*p *< 0.005) (Figure 3(h)). Similarly, at 7 days* BGLAP* showed a significant upregulation in Mg-63 cultures treated with 46.6 W/cm^2^ and 49 W/cm^2^ (*p <* 0.05) than those treated with 15.5 W/cm^2^, 23.3 W/cm^2^ and 31.1 W/cm^2^ (Figure 4(g)). The same trend was also observed in Saos-2; in particular,* BGLAP* expression was overexpressed with 49 W/cm^2^, which resulted is significant difference compared to 23.3 W/cm^2^ (*p <* 0.005) and 38.9 W/cm^2^ (*p <* 0.05) (Figure 4(h)). Finally, at 14 days* BGLAP *was significantly more expressed in both cell lines (Figures 5(g) and 5(h)) with 46.6 W/cm^2^ and 49 W/cm^2^ acoustic intensities (*p <* 0.05).

## 4. Discussion

In the present study, our aim was to investigate, for the first time by an* in vitro* model, if the impact of the dissipation of acoustic energies delivered outside the focal region during a FUS treatment might have any effect on Mg-63 and Saos-2 osteosarcoma cell viability and osteogenic differentiation. Osteosarcomas are characterized by different histologic subtypes that are composed of heterogeneous tumor cells [33]. Although osteosarcoma-derived cells are commonly used for osteoblast-like models, they differ in terms of proliferation kinetics and secretion of specific proteins. Mg-63 cells have fast proliferation rates and are a heterogeneous population showing both mature and immature phenotypes. On the contrary, Saos-2 cells are more mature and show a characteristic osteoblastic profile [34–36].

In line with these observations, in our study we focused our attention on non-thermal effects generated by FUS treatment; we devised a strategy of* in vitro* sonication using an experimental set-up that minimized the diffusion of thermal effects (max range was < 2°C), allowing the mechanical effects on propagate without appreciable decay within the sample. Firstly, the position of the focus was established by an interplay of simulations and experiments, where local temperature and acoustic pressure were verified. Secondly, we evaluated if the mechanical pressure exerted by the far field portion of the US field radiating from the focal lesion could give rise to cellular biological effects.

A relevant parameter that varies when an acoustic wave field crosses a tissue is acoustic pressure. In all experiments, the acoustic energy delivered to cells was transmitted by the far-field beam; this choice was made for two main reasons: first, the intensity profile of the far field is more homogeneous and easier to be accurately controlled than that of the near field; second, it has been experimentally verified on osteocytes that, at an axial distance beyond near field, acoustic energy can be transmitted more efficiently [37].

To investigate if different characteristics of osteosarcoma cell lines may have an influence on the response to FUS treatment, both Mg-63 and Saos-2 cells lines were exposed to different intensities of acoustic energy. Interestingly, both cell lines showed a different response based on the different level of acoustic intensities applied. The results suggest that a subpopulation of osteosarcoma cell lines, probably more prone to proliferation, survive in the presence of FUS mechanical and attenuated thermal effects and grow up to 14 days, expressing osteoblastic markers even 7 or 14 days after the treatment, even in experimental conditions that are usually applied for thermoablation (38.9-49 W/cm^2^). In our opinion, the attenuation of FUS acoustic intensities from the focal area (higher) to the “far field” (lower) zones may determine different osteosarcoma cell responses, which range from cell proliferation decrease (from 49 to 38.9 W/cm^2^) to improvement the maintenance of a subpopulation of living heterogeneous and immature cells (from 31.1 to 15.5 W/cm^2^) as demonstrated by the expression of early osteoblast markers* RUNX2 *and* ALPL*, which can clearly preserve bone tumor cells. In particular, the treatment of osteosarcoma cell lines with FUS energy higher that 38.9 W/cm^2^ showed a different response between Mg-63 and Saos-2 cells, reflecting their specific proliferating characteristics as described above.

Regarding the modulation of osteoblast gene expression markers, our data suggests that the two cell lines respond to FUS treatment in a different manner. Moreover, it would seem that FUS intensity of 38.9 W/cm^2^ might represent a cut-off below which surviving cells tend to become more undifferentiated or differentiated over time, depending also on their heterogeneity and immature phenotype. It is well known that abnormal expression of* RUNX2*,* ALPL,* and* BGLAP* determines impaired molecular and cellular functions in Mg-63 and Saos-2, but this phenomenon is different in the two osteosarcoma cell lines [34, 38]. It has recently been pointed out that* RUNX2* overexpression is a key pathological factor in osteosarcoma, by controlling different cancer-related genes, which correlates with metastasis and insufficient chemotherapy response [38, 39]. Since Mg-63 and Saos-2 osteosarcoma cells constitutively expressed* RUNX2 *at high levels throughout the cell cycle [38, 40, 41], we hypothesized that* RUNX2 *low expression, often observed in both cell types treated with FUS acoustic intensities higher than 38.9 W/cm^2^, might be mainly due to the destroying thermal and mechanical effects on cells, which seems directly proportional to the increase of the acoustic intensity applied.* ALPL *gene expression was downregulated in both cell models with FUS acoustic intensities lower than 41.2 W/cm^2^ at 24 h, but upregulated in Mg-63 with FUS acoustic intensities lower than 38.9 W/cm^2^ at 7 and 14 days, like an attempt to improve their differentiation phenotype. Conversely, Saos-2 showed an increasing early expression of* ALPL* gene, according to the increase of the acoustic intensities, maintaining higher than control over time, in almost all treatment conditions. Regarding* BGLAP*, it showed an expression lower than control at low intensity levels, similar in Mg-63 and Saos2, suggesting the maintenance of an undifferentiating phenotype under 38.9 W/cm^2^ even though the considered experimental times might be too early to detect appreciable BGLAP expression.

## 5. Conclusion

In* vitro* investigation of MgFUS effects on cancer cell lines has the potential to become a newly therapeutic strategy to enhance efficiency of* in vivo* cancer treatments. Our preliminary* in vitro* study suggested that osteosarcoma cell lines, treated with different FUS acoustic intensity levels, had a different ability to maintain or lose their differentiation state and relative proliferation capability. In particular, the FUS acoustic intensity to 38.9 W/cm^2^ might represent a cut-off, below which surviving cells tend to become more undifferentiated or differentiated over time, as demonstrated by cell viability and gene expression analysis.

Regarding this aspect, our results indicated that FUS treatments were able to induce many mechanotransduction effects on both cell lines. Briefly, we underlined as cell populations displayed a same regulation of* RUNX2*, while an important regulation of* ALPL* expression on Saos-2 cells was revealed after 24 h of treatments with high acoustic intensities and it is preserved over time with all acoustic intensity levels. Conversely,* ALPL* trend in Mg-63 was more fluctuating, decreasing with higher acoustic intensities, thus confirming an overall less differentiation level of Mg-63 compared to Saos2. Finally, also* BGLAP* expression seemed stimulated by higher acoustic intensities in both cell lines, suggesting that cells are more prone to differentiation in the zone near the treatment, but not in the same way.

The current data suggest that it would be important to investigate the response of each cell type or tumor tissue undergoing FUS treatment. According to this complexity and difference in responses, we think that further investigations in* in vivo* models of primary or metastatic bone tumor lesions would be mandatory before implementing clinical FUS treatment protocols, taking more into account areas surrounding the tumor lesion.

## Data Availability

Data are available on “https://figshare.com/s/2645fae70f9fdfab1c3c”.
